# Weather and Mortality

**Published:** 1902-08-23

**Authors:** 


### The Hospital

A Journal of

The Medical Sciences and Hospital Administration.

Vol. XXXII.—No. 830. Edited by Sir Henry Burdett, K.C.B., and Solomon C. Smith, M.D., M.R.C.P. [August 23, 1902.

### WEATHER AND MORTALITY.

The singularly cold, rainy and inclement weather of the past spring and early summer would have been expected by most people to be attended by an unusual mortality; and it is an agreeable surprise to discover, from the recently-issued quarterly report of the Registrar-General, that this apparently very reasonable expectation has not been fulfilled. The deaths registered during the quarter in England and Wales amounted to 132,241, and were in the proportion of 16·1 annually per thousand persons living, the average rate of the ten preceding second quarters having been 16·8; so that, in round numbers, the total number of deaths has been less than the average by some 23,000. The same tale is told by the application of the still more delicate test of infant mortality, which, measured by the proportion of deaths under one year of age to registered births, was 120 per thousand, the average in the ten preceding second quarters having been 125. The weather is described as having been changeable in April, but mainly fair and dry; in May it was for the most part cold and changeable, except during a short period which began about the end of the third week; and in June it was very cold and unseasonable until the last week of the quarter. At the Royal Observatory there were six rainy days in April, 25 in May, and 22 in June, the fall amounting to 0·42 of an inch, 3·33 inches, and 3·10 inches respectively. Judged by popular report, it is probable that the chief characteristic of the season, as it has been regarded by the ordinary citizen, has been the absence of anything which was even suggestive of "summer." On only two occasions, we believe, has the thermometer touched 80 degrees, and the abundant flight of straw hats which, in London, coincided with this achievement, disappeared well nigh as quickly as they came. Notwithstanding the almost total absence of anything which could be called high temperature, there have been numerous complaints of an oppressive condition of the atmosphere, productive of indisposition for active exertion; and it may perhaps be said that the chief characteristics of the quarter have been a persistence in the use of comparatively warm clothing, and a disinclination to submit to unnecessary physical fatigue under the name of pleasure. Is it possible that these two conditions, on the whole, and in their operation upon large numbers of people, have had some tendency towards the preservation of health?

The quarterly returns afford us little or no help towards the solution of the problems which they suggest, inasmuch as they give the actual causes of death only in relation to a small number of infectious diseases, the mortality from most of which, like that from general causes, has been somewhat below the average, a marked exception being afforded by small-pox, which was responsible for 1,054 deaths during the quarter. Taken together, the facts really seem to give some support to a set of doctrines and practices which might almost be described as "grandmotherly." Nature has made us take care of ourselves, and it seems as if we had not only reaped the reward, but as if we might derive therefrom a wholesome lesson for future guidance. It has been uncomfortable to do foolish things in the way of over fatigue or exposure, and so people have abstained from doing them. Old ladies and old gentlemen have not stopped to gossip at draughty street corners, and so they have escaped the pneumonia or the bronchitis, by which, in ordinary years, no inconsiderable toll is taken from their numbers. It is none the less noteworthy that the aged have not shared to the full in the increased duration of life of the general population, and it may therefore be presumed that their smaller power of resistance to low temperature has again displayed itself. Between the ages of one and sixty the mortality was at the annual rate of 8·4 per 1,000 of the estimated population at this group of ages, being 0·7 below the average of the ten preceding second quarters. Among persons aged 60 years and upwards the mortality was at the annual rate of 67·9 per 1,000 of the estimated population at this group of ages, the mean rate of the ten preceding second quarters having been 65·8. An increased death-rate of 2 1 per 1,000 would appear, therefore, to have been the tribute paid by old age to rain and low temperature; and this is certainly more intelligible than the general improvement, against which even the deaths from small-pox have been insufficient to turn the scale. We fear that the improvement cannot be attributed to any general advance in popular sanitation, for the latest reports of the medical officer of the Local Government Board are as full as usual of histories of fouled water and defective drainage in relation to the many local outbreaks of disease into which the inspectors of the department have been called upon to inquire. Nor have the conditions of the population, as far as we are aware, undergone any material change in the way of cleanliness, temperance, food supply, or general prosperity. We may expect that the diligence of Dr. Tatham, the untiring superintendent of statistics at the General Register Office, will by-and-by furnish us with more light upon the subject than we at present possess, and, in the meanwhile, all we can do is to accept our diminished death-rate thankfully, and to reflect upon the possibility that an inclement season may possibly sometimes prove to be a blessing in disguise.

